# Prevalence of latent tuberculosis infection in Asian nations: A systematic review and meta‐analysis

**DOI:** 10.1002/iid3.1200

**Published:** 2024-02-27

**Authors:** Abhigan B. Shrestha, Imran S. Siam, Jarin Tasnim, Abhinav Dahal, Poulami Roy, Sushil Neupane, Ashok Adhikari, Barsha Khanal, Rupesh Ghimirie, Dikshya Shrestha, Suju Bhattarai, Sajina Shrestha, Nischal Mainali, Yub Raj Sedhai, Uday Singh

**Affiliations:** ^1^ Department of Internal Medicine M Abdur Rahim Medical College Dinajpur Bangladesh; ^2^ Department of Internal Medicine Chattagram Maa O Shishu Medical College Chattogram Bangladesh; ^3^ Department of Internal Medicine Nepalese Army Institute of Health Sciences Kathmandu Nepal; ^4^ North Bengal Medical College and Hospital Siliguri West Bengal India; ^5^ Department of Internal Medicine Manipal College of Medical Sciences Pokhara Nepal; ^6^ Department of Internal Medicine Universal College of Medical Sciences Bhairawa Nepal; ^7^ Department of Internal Medicine Rangpur Medical College Rangpur Bangladesh; ^8^ Department of Internal Medicine Kist Medical College and Teaching Hospital Patan Nepal; ^9^ Department of Internal Medicine Kathmandu Medical College and Teaching Hospital Kathmandu Nepal; ^10^ Kathmandu Medical College and Teaching Hospital Kathmandu Nepal; ^11^ Division of Pulmonary Disease and Critical Care Medicine University of Kentucky College of Medicine Bowling Green Kentucky USA; ^12^ Department of Internal Medicine Nobel Medical College Biratnagar Nepal

**Keywords:** Asian, IGRA, latent tuberculosis, TST, tuberculosis

## Abstract

**Background:**

Tuberculosis (TB) is a serious public health concern around the world including Asia. TB burden is high in Asian countries and significant population harbor latent tuberculosis infection(LTBI).

**Aim:**

This systematic review and meta‐analysis aims to evaluate the prevalence of LTBI in Asian countries.

**Method:**

We performed a systematic literature search on PubMed, Embase, and ScienceDirect to identify relevant articles published between January 1, 2005, and January 1, 2023 investigating the overall prevalence of latent TB among people of Asia. Subgroup analysis was done for Asian subregions during the study period of 2011 to 2016 and 2017 to 2023, for tuberculin skin test (TST) and interferon gamma release assay (IGRA), respectively, as well as for QuantiFERON‐TB (QFT) and TSPOT TB tests. Der Simonian and Laird's random‐effects model was used to pool the prevalence of LTBI found using TST and IGRA.

**Result:**

A total of 15 studies were included after a systematic search from standard electronic databases. The analysis showed that the prevalence of latent TB in Asia was 21% (95% confidence interval [CI]: 19%–23%) and 36% (95% CI: 12%–59%) according to IGRAs and TSTs (cut off 10 mm) results, respectively. Based on IGRA, the prevalence of latent TB was 20% (95% CI: 13%–25%) in 2011 to 2016 and 21% (95% CI: 18%–24%) in 2017 to 2023. Using QFT, the prevalence was 19% (95% CI: 17%–22%) and using TSPOT, the prevalence was 26% (95% CI: 21%–31%). According to the United Nations division of Asia, the prevalence was higher for the Southern region and least for the Western region using TST and higher in the South‐Eastern region and least in the Western region using the IGRA test.

**Conclusion:**

Almost a quarter of the Asian population has LTBI. Its diagnosis often poses a diagnostic challenge due to the unavailability of standard test in certain areas. Given this prevalence, a mass screening program is suggested with the available standard test and public awareness along with anti‐TB regimen should be considered for individuals who test positive. However, for it to be implemented effectively, we need to take the affordability, availability, and cost‐effectiveness of such interventions into account.

## 1 INTRODUCTION

As defined by the World Health Organization (WHO), latent tuberculosis infection (LTBI) is a state without any clinical symptoms of active tuberculosis (TB), despite persistent immune stimulation by *Mycobacterium tuberculosis* (Mtb).^1^ Even though it may not seem to pose an immediate threat, approximately 10% of healthy individuals with latent TB are at risk of developing active TB, which carries about a 50% mortality rate without treatment, in their lifetime. According to a study in 2019, it is estimated that about a quarter of the world's population may be latently infected with TB, with Southeast Asia leading in prevalence.^2^ Six countries with a high global TB burden—Bangladesh, the Republic of Korea, India, Indonesia, Myanmar, Thailand, and the Democratic People's Republic of Korea, all belong to this region.^3^ Factors predisposing to lung diseases, like surging tobacco use, occupational lung diseases, indoor pollutants from wood and dung used as cooking fuel and alarming outdoor pollution in Asia, can be attributed to the high prevalence of TB in this region.^4^, ^5^, ^6^

The WHO aims to reduce the incidence rate and absolute death toll caused by TB to 90% and 95%, respectively, by 2035 compared to the 2015 baseline. This has been significantly impacted by the coronavirus disease 2019 pandemic, with a limitation in reported cases, as evidenced by a 3.6% increase in incident TB cases between 2020 and 2021 alone, reversing the years of already slow declines and hindering efforts to achieve the goal, suggesting a concerning increase in undiagnosed, untreated, and more community transmission of the infection.^3^, ^7^ The statistics are even more terrifying, showing that the majority of global incident cases in 2021 were from Asia alone even with the large absolute reduction in reported cases of TB were from India, China, Indonesia, Philippines, Myanmar, Bangladesh, and Pakistan.^3^ Even if the transmission had been completely interrupted, there would still be more than 100 cases per million population in 2050 by reactivation and relapse of old infections which highlights the utmost need to neutralize the reservoir of latent infection along with its active transmission.^7^, ^8^

The recent methodologies for diagnosing LTBI include the tuberculin skin test (TST) and the interferon‐gamma release assay (IGRA) supplemented by physical examinations, chest X‐rays, exposure and medical history.^1^, ^3^ Due to low cost and ease of use, TST has been traditionally used as a screening tool for LTBI, which detects antigen specific T‐cell response amongst exposed contacts.^9^, ^10^ Evidence shows that among the Bacillus Calmette–Guérin (BCG)‐vaccinated populations and regions with frequent nontuberculous mycobacteria (NTM) exposure, recent techniques with IGRAs have shown a higher sensitivity and specificity with higher rates of long‐term development of active TB among positive IGRA cases.^11^, ^12^ The importance of LTBI on disease natural history and its major impact on the population in Asia shows that TB cannot be defeated without understanding how to eliminate it in this region.^13^ A number of studies have been done to estimate the global prevalence of LTBI using both IGRA and TST^2^, ^14^; however, the exact burden of LTBI in the Asia region must be thoroughly studied using available standard tests to achieve global TB elimination by 2035.

## 2 METHODS

### 2.1 Search strategy and selection criteria

This study adheres to best practices for the conduct of systematic reviews and meta‐analyses, in addition to the Preferred Reporting Items for Systematic Reviews and Meta‐analyses (PRISMA) guidelines.^2^, ^15^, ^16^, ^17^ The protocol was registered on PROSPERO CRD42023439932.

All authors independently searched the following electronic databases: PubMed, ScienceDirect, and Embase. The search was conducted to identify relevant articles published between January 1, 2005 and January 1, 2023. The search period, beginning from 2005, reflects the widespread availability of commercial IGRAs in the market, aiming to provide an updated analysis with the latest surveys. The QuantiFERON‐TB (QFT) and QFT Gold (QFT‐G) tests were introduced in 2001 and 2005, respectively.^18^, ^19^ Similarly, the T‐SPOT.TB test was introduced in 2004. It was approved for use in China in 2010.^20^ Moreover, it has been approved in numerous countries such as Taiwan and Singapore which are included in our study.^21^

The search strategy encompassed both free text and medical subject heading terms, and the keywords were searched in the title, abstract, and field keywords of the articles using appropriate Boolean operators (AND/OR) including all Asian nations and available standard tests; “TST,” “Mantoux,” and “Interferon gamma release assay,” “QuantiFERON‐TB,” “TSPOT.” The objective was to identify studies related to the prevalence of latent TB and its diagnosis using tuberculin tests, IGRAs, and enzyme‐linked immunospot assays. The detailed search strategy can be found in the Supporting Information S1. Language barrier was not considered during the screening and selection process.

### 2.2 Study selection

#### 2.2.1 Inclusion criteria

1. Studies reporting on the prevalence of LTBI diagnosed with IGRA and/or 10 mm cut‐off—TST.
2. Studies published between 2005 and 2023.
3. Studies with a sample size of at least 100 participants to minimize selection bias from small studies.
4. Studies that provide data on the prevalence of LTBI available to be stratified by country, specifically including data from Asian countries.
5. Both retrospective and prospective studies were included.

#### 2.2.2 Exclusion criteria

1. Reviews or meta‐analyses.
2. Case reports or case series.
3. Cost‐effectiveness analyses.
4. Studies involving non‐human populations.
5. Studies where the study population is not tested with either TST or IGRA (QFT‐G, QFT‐GIT, QFT‐Plus, T‐SPOT.TB).
6. Studies that include interventions before testing that could affect the results.
7. Studies that select the study population based on culture or radiographic findings indicate infection with any mycobacteria as a mandatory criterion.
8. Studies that select the study population based on specific results of TST or IGRA as a mandatory criterion.
9. Studies that focus on high‐risk groups (e.g., drug users, prison inmates, psychiatric patients, indigenous populations, healthcare workers, medical students with clinical exposure, homeless individuals, human immunodeficiency virus (HIV)‐positive patients, prisoner, contact investigations, refugee/migrant screening, organ transplant recipients, sarcoidosis, silicosis, miners, patients with end‐stage renal disease or undergoing dialysis, patients with inflammatory‐mediated disease, patients with diabetes mellitus, individuals taking immunosuppressant agents, individuals with TB contact).
10. Studies with participants under the age of 18.

In addition to the above exclusion criteria, we also decided to exclude studies which used a TST cut‐off of 5 mm as 10 mm cut‐off are more reliable to diagnose LTBI in moderate to low‐risk populations while 5 mm cut‐off are used to diagnose LTBI in high‐risk individuals such as immunocompromised and HIV‐infected individuals. By doing so, we aim to study a broader and more generalized prevalence of LTBI among Asian countries.

After removing duplicates, all investigators independently screened the titles and abstracts initially based on the inclusion and exclusion criteria. If the inclusion criteria were met, the full‐text were then reviewed. Moreover, we also reviewed previously published systematic reviews relevant to our topic.

Any discrepancies or disagreements were resolved through discussion or consultation with the first author and supervisors if necessary.

### 2.3 Quality assessment

The risk of bias was assessed using the Newcastle‐Ottawa Scale (NOS) for observational studies. NOS is an important tool used for evaluating the quality of observational studies to be included in a systematic review. Three major parameters were checked: selection, comparability, and the outcome. The step was proceeded by two authors independently after screening the full text. The mean score of the two authors was taken into account for the decision. Studies were addressed for its biases accordingly; high (<5 stars), moderate (5–7) stars or low risk of bias (≥8 stars).

### 2.4 Data extraction

We conducted a comprehensive review of the selected studies and extracted pertinent information including the first author's name, publication year, study design, study date, description of the study population, age, exclusion criteria, as well as data on enrolled participants. In cases where data for specific subgroups were unavailable, we utilized enrollment data for the overall study population. Additionally, we collected information on TB verification methods, sample size, the specific type of tests used (e.g., QFT, T‐SPOT.TB, TST), and the cut‐off values employed for TST. For this, a dual‐review process was employed, where initially one author extracted relevant data from each selected study and subsequently, a second author independently verified this extracted data for accuracy.

### 2.5 Statistical analysis

Statistical analyses were conducted to interpret the data extracted from the selected studies. The calculations including mean, median, standard deviation and organizing data for heterogeneity assessments and prevalence estimates were conducted in Microsoft Excel 2016 (Microsoft Corp.). The data from the Excel sheet were extracted to STATA version 17.0 (StataCorp). We pooled the prevalence of LTBI based on the proportion of individuals who returned a positive result on either IGRA and TST. This was done with Der Simonian and Laird's random‐effects model due to different population and demographic settings across studies.^22^ With this difference, we anticipated considerable heterogeneity among the included studies. Heterogeneity among studies was examined using the Cochrane *Q* test and *I* ^2^ statistics. Substantial heterogeneity was considered for a value of *I* ^2^ > 75%. For the determination of the source of heterogeneity, further subgroup analysis was done: Asian region and study period (2011–2016, 2017–2023) for TST and IGRA, and QFT and TSPOT for IGRA. Publication bias was noted using the funnel plot of the overall effect size against its standard error with potential publication bias filled using STATA trim and fill command. For the small study effect size, Egger's regression test was performed. *p* < .1 was regarded as statistically significant for publication bias.

## 3 RESULTS

### 3.1 Study selection and characteristics

**Figure 1:**
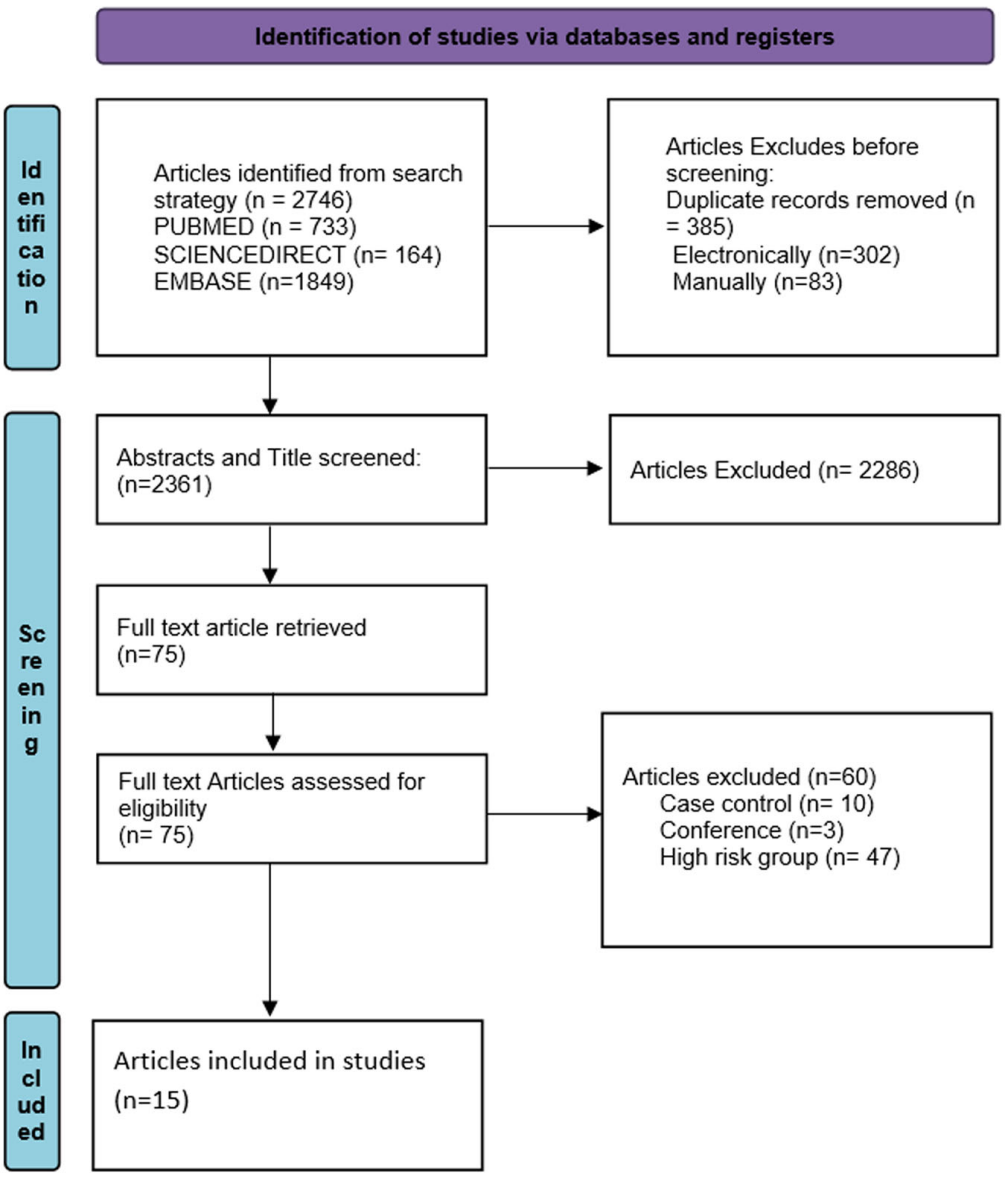
Preferred Reporting Items for Systematic Reviews and Meta‐analyses flowchart for the study selection process.

**Figure 2:**
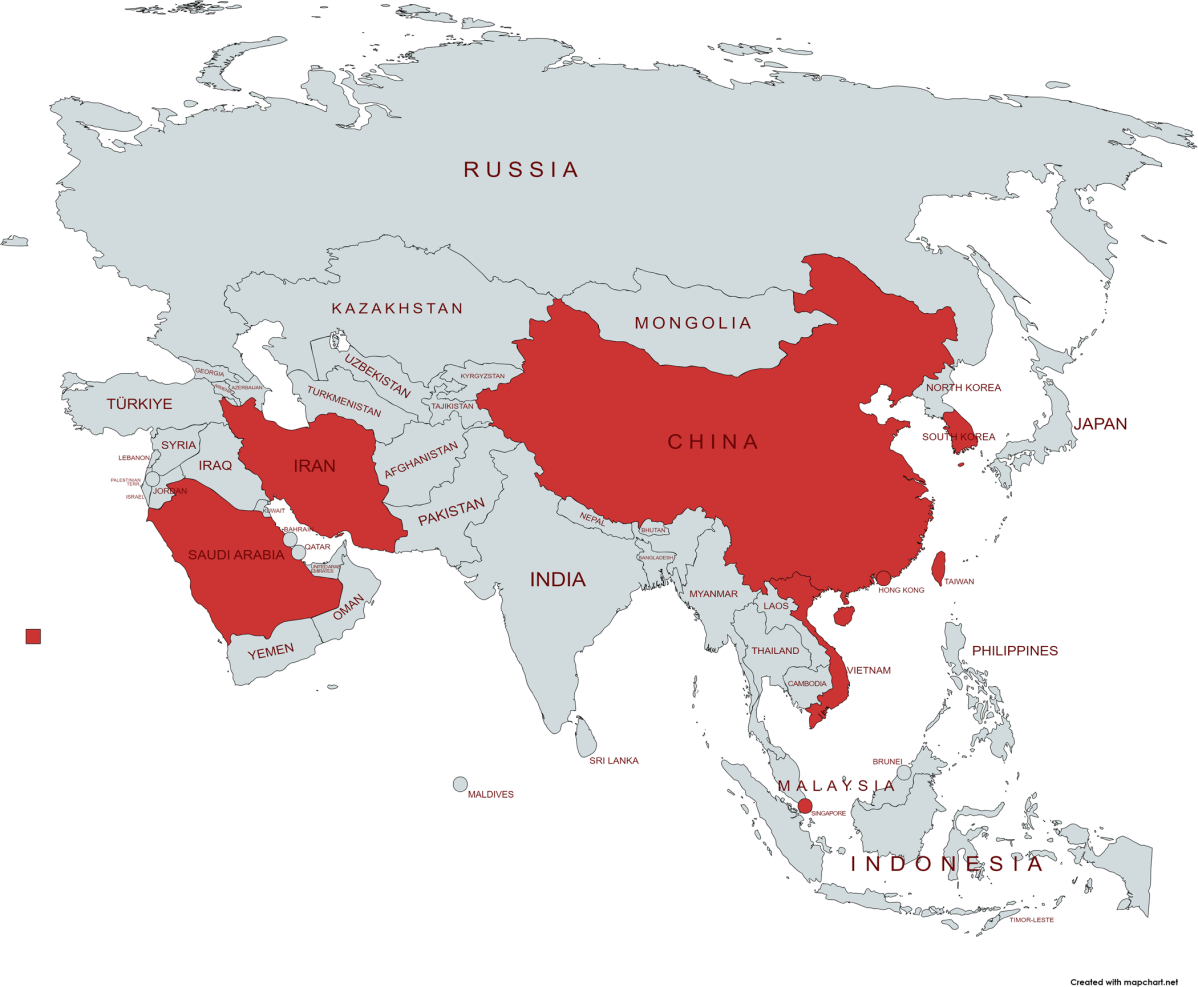
Included Asian countries with latent tuberculosis infection for calculation.

A total of 15 studies were included following a systematic search of standard electronic databases.^23^, ^24^, ^25^, ^26^, ^27^, ^28^, ^29^, ^30^, ^31^, ^32^, ^33^, ^34^, ^35^, ^36^, ^37^ The PRISMA flow diagram for the study selection process is shown in Figure 1. Altogether, 144,561 participants were included in this meta‐analysis. All articles were published between 2006^30^ and 2023.^26^ As per the United Nations (UN) subdivision of Asia, two articles were from Western Asia,^28^, ^36^ 10 articles from East Asia,^24^, ^26^, ^27^, ^30^, ^31^, ^32^, ^33^, ^34^, ^35^, ^37^ two articles from Southeast Asia,^25^, ^29^ and one article from Southern Asia.^23^ As per the WHO subdivision of Asia,^38^ three articles were from the Eastern Mediterranean Region^23^, ^28^, ^36^ and 12 articles from Western Pacific Region.^24^, ^25^, ^26^, ^27^, ^29^, ^30^, ^31^, ^32^, ^33^, ^34^, ^35^, ^37^ Country wise, five articles from China,^24^, ^31^, ^32^, ^33^, ^34^ three articles from South Korea,^26^, ^27^, ^35^ two articles from Saudi Arabia,^28^, ^36^ and each from Singapore,^29^ Hong Kong,^30^ Iran,^23^ Taiwan,^37^ and Vietnam^25^ were represented in this study (Figure 2). The mean age among reported studies ranged from 19.7^31^ to 82 years.^30^ Male participants comprised 30% of the total in the reported studies. The detailed baseline characteristics of the included studies is provided in Table 1.

**Table 1:** Baseline characteristics of included studies.

Study	Country; TSPOT and QFT		Sample tested positive by TST	Total population	Sample tested positive by LTBI	Total population
Khoufi et al. (2021)	Saudi Arabia	QFT	NA	NA	19	98
Hwang et al. (2018)	Korea	QFT	NA	NA	4519	23,807
Liu et al. (2017)	China	QFT	NA	NA	524	2185
Balkhy et al. (2017)	Saudi Arabia	QFT	127	1369	124	1369
Chen et al. (2015)	China	QFT	NA	NA	1060	5305
Besharat et al. (2011)	Iran		590	1212	NA	NA
Wu et al. (2011)	China	TSPOT	455	907	278	907
Wu et al. (2009)	China	TSPOT	41	100	21	100
Jung et al. (2012)	Korea	QFT	23	153	8	153
Lin et al. (2019)	Taiwan	QFT	NA	NA	81	258
Kim et al. (2023)	Korea	QFT	NA	NA	20,430	101,373
Marks et al. (2018)	Vietnam	QFT	NA	NA	486	1319
Zhao et al. (2011)	China	TSPOT	116	899	223	899
Yeung et al. (2006)	Hong Kong		2640	3682	NA	NA
Yap et al. (2018)	Singapore	QFT	NA	NA	213	1682

Abbreviations: LTBI, latent tuberculosis infection; QFT, QuantiFERON‐TB; TST, tuberculin skin test.

Among these studies, 10 studies used QFT‐G (*n* = 137,533),^24^, ^25^, ^26^, ^27^, ^28^, ^29^, ^34^, ^35^, ^36^, ^37^ seven studies used TST10 mm (*n* = 8401),^23^, ^27^, ^28^, ^30^, ^31^, ^32^, ^33^ three studies used T‐SPOT.TB (*n* = 1773).^31^, ^32^, ^33^ In total, 13 studies used IGRA (either QFT or TSPOT) (*n* = 139,455).^24^, ^25^, ^26^, ^27^, ^28^, ^29^, ^31^, ^32^, ^33^, ^34^, ^35^, ^36^, ^37^

### 3.2 Risk of Bias Assessment

All studies were low to moderate risk and included in the analysis (Supporting Information S2).

### 3.3 Pooled prevalence of latent TB and subgroup analysis

**Figure 3:**
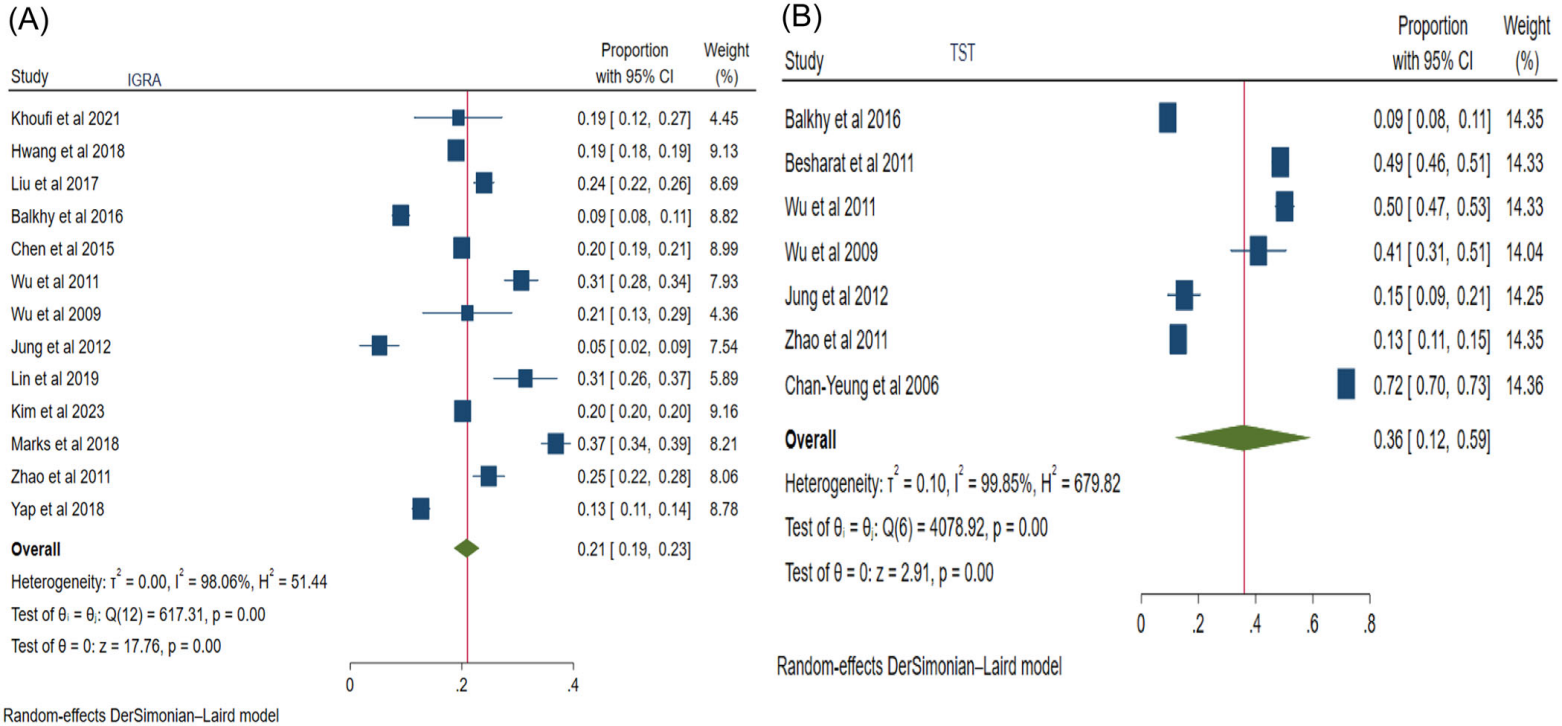
(A) Forest plot showing the prevalence of LTBI in Asia using IGRA. (B) Forest plot showing the prevalence of LTBI in Asia using TST. IGRA, interferon‐gamma release assay; LTBI, latent tuberculosis infection; TST, tuberculin skin test.

The prevalence of LTBI in Asia was 21% (95% confidence interval [CI]: 19%–23%); *I* ^2^: 98.06% and 36% (95% CI: 12%–59%); *I* ^2^: 99.85% according to IGRA and TST (cut off 10 mm) results, respectively (Figure 3A,B).

**Figure 4:**
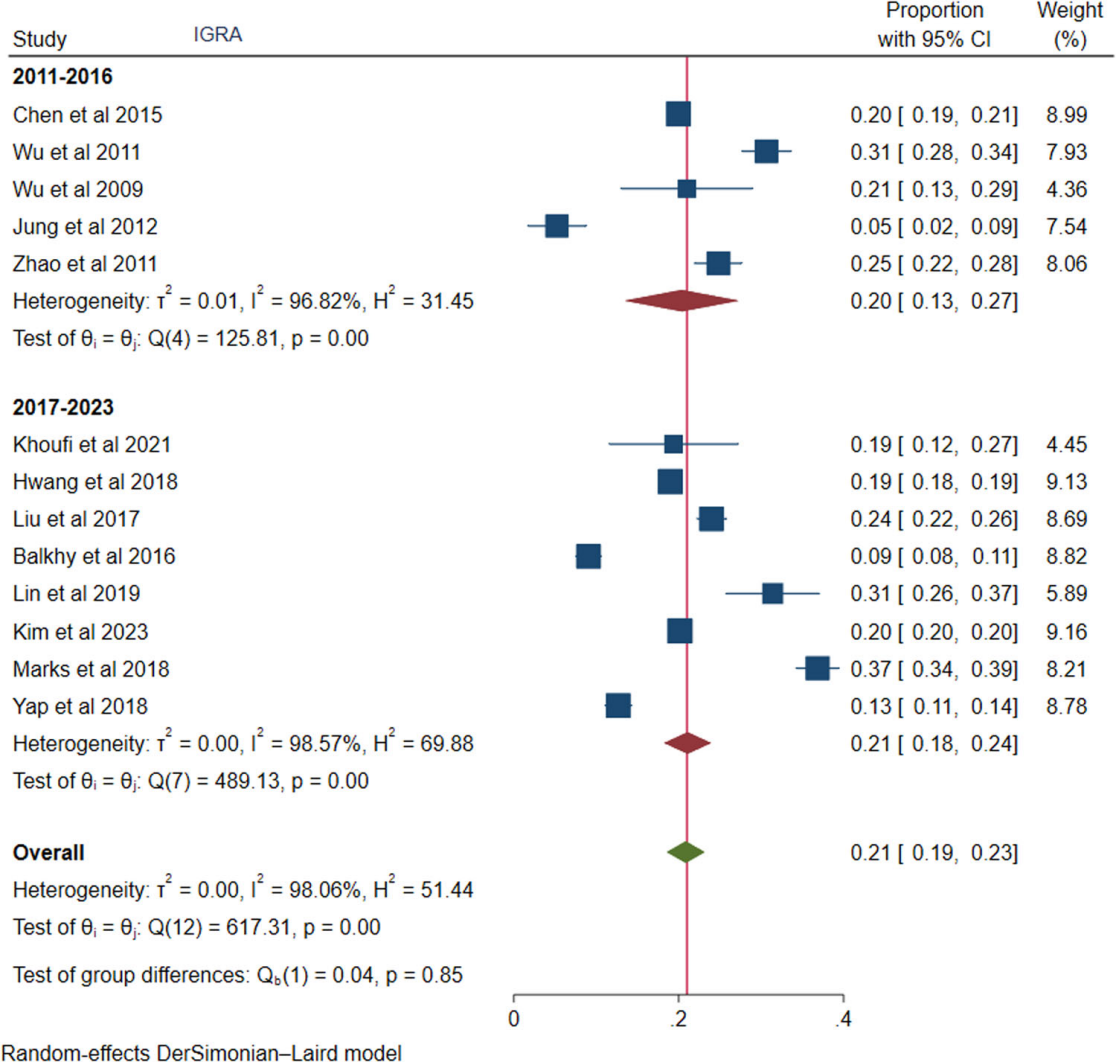
Forest plot showing subgroup analysis for the prevalence of LTBI in Asia for Time trend. CI, confidence interval; IGRA, interferon‐gamma release assay; LTBI, latent tuberculosis infection.

Based on the IGRA result, the prevalence of Latent TB was 20% (95% CI: 13%–25%); *I* ^2^: 96.82% in 2011 to 2016 and 21% (95% CI: 18%–24%); *I* ^2^: 98.57% in 2017 to 2023 (Figure 4).

**Figure 5:**
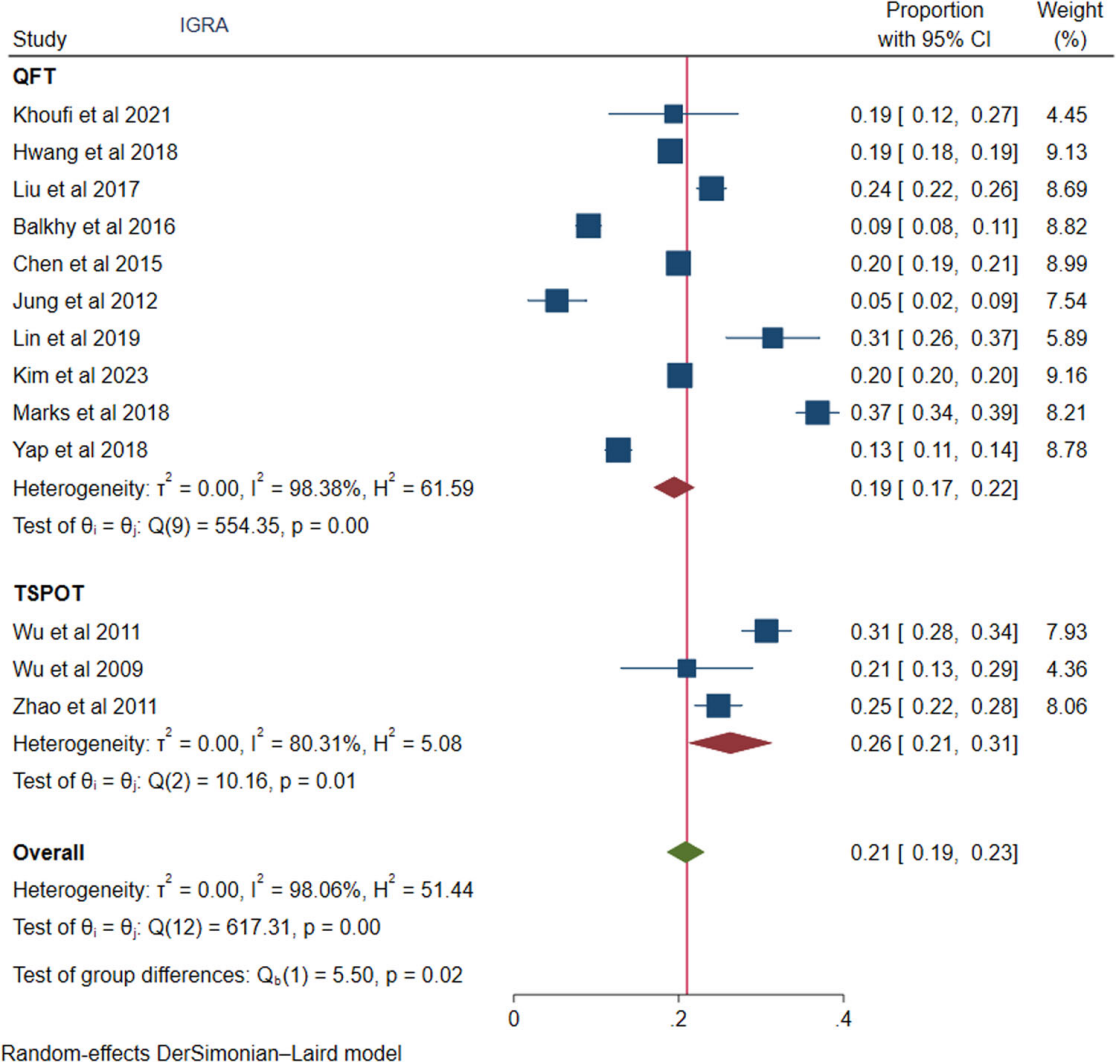
Forest plot showing subgroup analysis for the prevalence of LTBI in Asia for QFT and TSPOT. CI, confidence interval; LTBI, latent tuberculosis infection; QFT, QuantiFERON‐TB.

Using QFT, the prevalence was 19% (95% CI: 17%–22%); *I* ^2^: 98.38% and using T‐SPOT.TB, the prevalence was 26% (95% CI: 21%–31%), *I* ^2^: 80.31% (Figure 5).

### 3.4 Region‐wise analysis

According to the UN subdivision of Asia, the prevalence of latent TB was highest in the Southern region using TST10 mm and in the South‐Eastern region using IGRA (Table 2).

**Table 2:** Subgroup analysis for the prevalence of LTBI in Asia according to UN subdivision.

Asia subdivision	Prevalence using IGRA	Prevalence using TST
Eastern	21% (95% CI: 20%–23%)	38% (95% CI: 9%–68%)
Southern	–	49% (95% CI: 46%–51%)
Western	13% (95% CI: 3%–23%)	9% (95% CI: 8%–11%)
South‐Eastern	25% (95% CI: 1%–48%)	–

Abbreviations: CI, confidence interval; IGRA, interferon‐gamma release assay; LTBI, latent tuberculosis infection; TST, tuberculin skin test; UN, United Nations.

### 3.5 Publication bias and sensitivity analysis

The funnel plot analysis with trim and fill did not show any additional studies. The regression‐based Egger's test indicated no influence of small study effect size.

For IGRA, omitting one study at a time showed comparable outcomes, however, the prevalence ranged 19%–22%, whereas for TST10 mm ranged from 29% to 40% (Supporting Information S3).

## 4 DISCUSSION

To our knowledge, this is the first meta‐analysis shedding light on the prevalence of LTBI in Asia. The prevalence of latent TB was measured using the IGRA test and TST. IGRA shows a prevalence of 21% and TST, on the other hand, shows a 36% prevalence. Subgroup analysis revealed a prevalence of 19% for QFT and 21% for TSPOT.

This meta‐analysis emphasized the diagnostic role of IGRA and TST in identifying latent TB infection within the community. A total of 139,455 IGRA tests were done out of which 27,986 were positive. The Southeast Asia region has reported a significant prevalence of latent TB infection (31%) in comparison to the Western Pacific region (28%) and other regions (11%–22%).^14^ Early as well as accurate diagnosis will help in meeting the WHO's END TB strategy, a global annual reduction of about 14% is enough to bring down the huge caseload by 2050.^7^ The treatment of latent TB to prevent the development of active TB is a crucial part of WHO's END TB strategy.^1^ The diagnosis of LTBI often poses a diagnostic challenge due to the unavailability of IGRA or TST in certain areas. However, the latest 2018 WHO guidelines proposed the initiation of LTBI treatment even before the LTBI testing in the high‐risk population.^1^ Cambodia, Myanmar, and India are the regions where TST is the only mode of LTBI diagnosis whereas Bangladesh, Hong Kong, Indonesia, Malaysia, Philippines, and Thailand are regions where both TST and IGRA is performed. However, due to cost issues TST is mostly done for public usage while IGRA is used majorly in the private setup and for research purposes.^39^ Both the LTBI detection tests are based on the principle of memory T cells from previous infections; thus, a positive test does not invariably indicate viable infection. QFT which is an upgraded version of IGRA, has been claimed to have a higher efficacy in detecting active infection, as they target both the CD4 and CD8 cells.^40^, ^41^

TST as well as IGRA, which work on the principle of cell‐mediated immunity, are the currently accepted methods for LTBI diagnosis.^42^, ^43^

The accuracy of TST is often compromised by confounding factors such as BCG vaccination and NTM infections. To overcome these limitations, IGRAs incorporating the region of difference‐1 antigen‐specific to Mtb have been developed. These IGRAs claim to possess higher specificity than TST.^12^ However, the lack of a definitive gold standard has resulted in limited data regarding the diagnostic performance of these tests in identifying LTBI.

The principal aim of LTBI testing is to discern individuals who are susceptible to progressing to active TB in subsequent instances. Extensive research has underscored the restricted prognostic capability of both these assays, which manifest commensurate efficiency and can be employed interchangeably.^44^

Nevertheless, it is imperative to acknowledge that neither of these assays can accurately ascertain infection status or serve as a dependable determinant for instigating preventative therapy among afflicted individuals.

Although both these tests are instrumental in detecting LTBI, they also share some limitations. The local challenges with TST include shortage of reagents, increasing dropout rates and low specificity to detect active infection. The challenges with IGRA include high cost, limited ability, technical complications, lack of adequate infrastructure, indeterminate sources, and lack of specificity. The 2018 guidelines recommend isoniazid monotherapy in LTBI. However, low compliance in Asian countries implemented a short duration two course regimen.^1^

Meeting the WHO End TB strategy might seem like a gigantic task, nevertheless proper screening, addressing unmet needs and prioritizing high‐risk groups will help in the achievement of the goal.

The TST has the potential to yield false‐positive results attributed to previous BCG vaccination or sensitization to NTM. This may explain why we have seen the greater prevalence of LTBI by TST. Conversely, false‐negative outcomes can arise from factors including immunosuppression (such as HIV or active TB), gradual decline of natural immunity, and technical limitations encompassing variations in interpretation among readers. In contrast, the IGRA is not susceptible to false‐positive results due to prior BCG vaccination. Nonetheless, false‐negative outcomes may occur in IGRA due to immunosuppression (such as HIV or active TB) and technical variability.^45^

In high TB burden countries with limited resources, the choice of diagnostic tests for LTBI should take into account factors such as cost, logistical feasibility, target population, and individual preferences. It is recommended that the TST continue to be used as the main method for LTBI diagnosis due to its affordability, ease of administration, absence of specialized expertize requirement, availability of standardized laboratories, and utilization of venepuncture. This recommendation aligns with the WHO's first comprehensive guidelines on LTBI management, which conditionally endorse the use of TST for diagnosing LTBI in low‐ and middle‐income countries.^46^

In their study, Cohen et al.^2^ found the global prevalence of latent TB to be 24.8% and 21.2% according to IGRA and TST results, respectively. However, a change in trend was seen when latent TB prevalence was studied in the Asian population. The IGRA and TST recorded prevalence to be 21% and 36%. There was an observed increase in latent TB prevalence, rising from 20% in 2011 to 2016 to 21% in 2017 to 2023. Although this change might appear to be minimal, considering the dynamic population size, the number of new cases have seen a sharp rise. The reasons for this rise might be implied to factors like increasing caseload among the elderly, nonadherence to treatment and inadequate screening in high‐risk areas.

Public awareness plays an important role in combating the problem of LTBI in the community. It makes people aware about the risk factors, diagnosis as well as the treatment of LTBI. It encourages people to adhere to their treatment regimen and also helps to destigmatize TB in the community.

## 5 LIMITATIONS

Since this study focuses on the Asian population, including a broader spectrum of the population would help us in getting a larger view of the problem in hand. Improper generalization of subgroups might lead to distorted results. Moreover, not all Asian countries were represented in the meta‐analysis, potentially affecting the generalization of our findings across the entire Asian continent. Also, like other meta‐analysis, this study was also vulnerable to publication bias which might lead to overestimation of the true effect size. Heterogeneity might result in pooling of data, which in turn causes limitation of generalization of data. Making inferences at the individual level based on group‐level findings can lead to an ecological fallacy, in which the conclusions drawn for a particular group may not hold true for individual cases. Moreover, in our analysis, there is potential for false positive results from the diagnostic tests used in the included studies, namely IGRA and TST. TST may yield false positives in BCG‐vaccinated individuals as well as in those individuals exposed to NTM. Similarly, while IGRAs are less prone to false positives from BCG vaccination, they may show false positive results as well. Thus, these limitations must be kept in mind while interpreting the results of a meta‐analysis.

## 6 CONCLUSION

Almost a quarter of the Asian population harbors LTBI. Its diagnosis often poses a diagnostic challenge, primarily due to the unavailability of standard tests in certain areas. However, with this prevalence, a mass screening program is suggested with the available standard test. Moreover, public awareness initiatives along with anti‐TB regimens should be considered for individuals who test positive.

## AUTHOR CONTRIBUTIONS

**Abhigan B. Shrestha** and **Sajina Shrestha**: Conceptualization. **Abhigan B. Shrestha**: Formal analysis and investigation. **Poulomi Roy**, **Ashok Adhikari**, **Abhinav Dahal**, **Sushil Neupane**, **Imran S. Siam**, **Jarin Tasnim, Barsha Khanal**: Writing—original draft preparation. **Suju Bhattarai**, Rupesh Ghimire, Dikshya Shrestha, **Sajina Shrestha**, **Nischal Mainali**, **Yub Raj Sedhai**, **Uday Singh**: Writing—review and editing. **Yub Raj Sedhai**: Supervisor. None of the authors were involved in funding acquisition and all the authors were involved in the methodology.

## CONFLICT OF INTEREST STATEMENT

The authors declare no conflict of interest. The abstract has been submitted to the ATS24 conference.

## Supporting information

S1: search strategy for included articles.

S2: Risk of bias assessment using New Castle Ottawa Scale for observational studies.

S3: Publication bias, Regression based Eggers’ test and sensitivity analysis for IGRA and TST.

## Data Availability

Data are available from the corresponding author on request.
