# Supplementary material for: Prevalence of latent tuberculosis infection in Asian nations: A systematic review and meta‐analysis
Source: Immun Inflamm Dis. 2024 Feb 27;12(2):e1200. doi: 10.1002/iid3.1200 (PMC10898208; doi:10.1002/iid3.1200)
Supplement: Supplementary file 1 — S1: search strategy for included articles. [file IID3-12-e1200-s003.docx]

Search strategy

**#1 (((((((((((((((((((((((((((((((((((((((((((((((china) OR (india)) OR (indonesia)) OR (pakistan)) OR (bangladesh)) OR (japan)) OR (philippines)) OR (vietnam)) OR (turkey)) OR (iran)) OR (thailand)) OR (myanmar)) OR (south korea)) OR (iraq)) OR (afghanistan)) OR (saudi arabia)) OR (uzbekistan)) OR (malaysia)) OR (yemen)) OR (nepal)) OR (north korea)) OR (sri lanka)) OR (kazakhstan)) OR (syria)) OR (cambodia)) OR (jordon)) OR (azerbaijan)) OR (united arab emirates)) OR (tajikistan)) OR (israel)) OR (laos)) OR (lebanon)) OR (kyrgyzstan)) OR (turkmenistan)) OR (singapore)) OR (oman)) OR (sate of palestine)) OR (kuwait)) OR (georgia)) OR (mongolia)) OR (armenia)) OR (qatar)) OR (bahrain)) OR (timor-leste)) OR (cyprus)) OR (bhutan)) OR (maldives)) OR (brunei)**

**#2 ((((((((TST) OR (tuberculin skin test)) OR (tuberculin test)) OR (interferon-gamma release assay)) OR (interferon gamma)) OR (IGRA)) OR (QFT)) OR (t-spot.tb)) OR (enzyme linked immunospot assay)**

**#3 ((LTBI) OR (latent tuberculosis)) OR (latent mycobacter*)**

**#4 ((prevalance) OR (proportion)) OR (rate)**

(((((prevalance) OR (proportion)) OR (rate)) AND (((LTBI) OR (latent tuberculosis)) OR (latent mycobacter*))) AND (((((((((TST) OR (tuberculin skin test)) OR (tuberculin test)) OR (interferon-gamma release assay)) OR (interferon gamma)) OR (IGRA)) OR (QFT)) OR (t-spot.tb)) OR (enzyme linked immunospot assay))) AND ((((((((((((((((((((((((((((((((((((((((((((((((china) OR (india)) OR (indonesia)) OR (pakistan)) OR (bangladesh)) OR (japan)) OR (philippines)) OR (vietnam)) OR (turkey)) OR (iran)) OR (thailand)) OR (myanmar)) OR (south korea)) OR (iraq)) OR (afghanistan)) OR (saudi arabia)) OR (uzbekistan)) OR (malaysia)) OR (yemen)) OR (nepal)) OR (north korea)) OR (sri lanka)) OR (kazakhstan)) OR (syria)) OR (cambodia)) OR (jordon)) OR (azerbaijan)) OR (united arab emirates)) OR (tajikistan)) OR (israel)) OR (laos)) OR (lebanon)) OR (kyrgyzstan)) OR (turkmenistan)) OR (singapore)) OR (oman)) OR (sate of palestine)) OR (kuwait)) OR (georgia)) OR (mongolia)) OR (armenia)) OR (qatar)) OR (bahrain)) OR (timor-leste)) OR (cyprus)) OR (bhutan)) OR (maldives)) OR (brunei))
