# Supplementary material for: Prevalence of latent tuberculosis infection in Asian nations: A systematic review and meta‐analysis
Source: Immun Inflamm Dis. 2024 Feb 27;12(2):e1200. doi: 10.1002/iid3.1200 (PMC10898208; doi:10.1002/iid3.1200)
Supplement: Supplementary file 2 — S2: Risk of bias assessment using New Castle Ottawa Scale for observational studies. [file IID3-12-e1200-s001.docx]

| **Studies** | **Seletion1** | **Seletion2** | **Comparability1** | **Comparability2** | **Outcome1** | **Outcome2** | **a1** | **a2** |
| --- | --- | --- | --- | --- | --- | --- | --- | --- |
| Khoufi et al | ** | ** | * | * | ** | ** | 5* | 5* |
| Hwang et al | ** | ** | ** | ** | * | * | 5* | 5* |
| Liu et al | ** | ** | ** | * | * | * | 5* | 4* |
| Balkhay et al | ** | ** | ** | ** | ** | * | 6* | 5* |
| Chen et al | ** | ** | ** | ** | ** | ** | 6* | 6* |
| Besharat et al | ** | ** | ** | ** | * | * | 5* | 5* |
| Wu et al | ** | ** | ** | ** | * | ** | 5* | 6* |
| Wu et al | * | * | ** | ** | *** | *** | 6* | 6* |
| Jung et al | ** | ** | * | * | ** | ** | 5* | 5* |
| Lin et al | ** | ** | ** | ** | ** | ** | 6* | 6* |
| Kim et al | ** | ** | ** | ** | ** | ** | 6* | 6* |
| Marks et al | ** | ** | ** | ** | ** | * | 6* | 5* |
| Zhao et al | ** | ** | ** | ** | *** | ** | 7* | 6* |
| Chan-Yeung et al | ** | ** | * | * | ** | ** | 5* | 5* |
| Yap et al | ** | ** | * | ** | ** | ** | 5* | 6* |
