# Supplementary material for: Prevalence of latent tuberculosis infection in Asian nations: A systematic review and meta‐analysis
Source: Immun Inflamm Dis. 2024 Feb 27;12(2):e1200. doi: 10.1002/iid3.1200 (PMC10898208; doi:10.1002/iid3.1200)

1. Publication bias for IGRA and TST


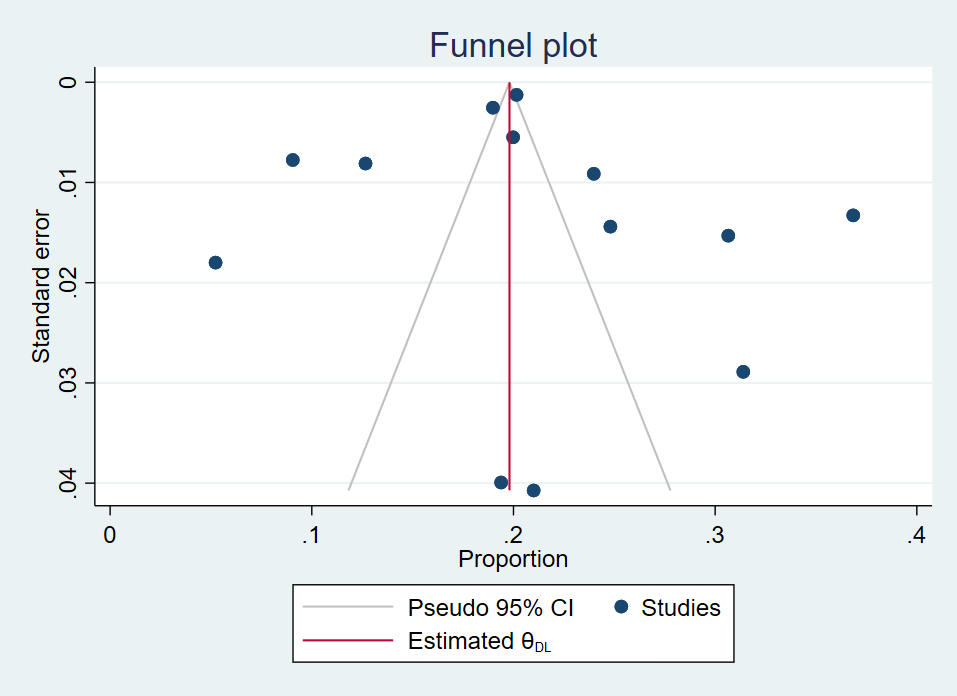

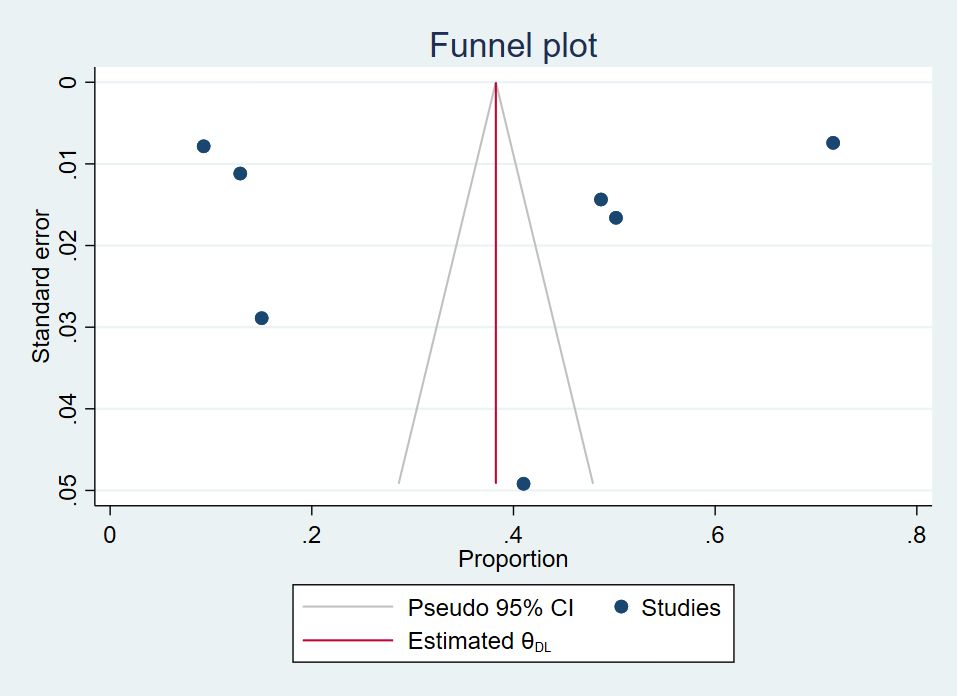


1. Regression based Eggers

TST

: beta1 = 0; no small-study effects

beta1 = -0.63

SE of beta1 = 9.239

z = -0.07

Prob > |z| = 0.9453

IGRA

beta1 = 0; no small-study effects

beta1 = 1.08

SE of beta1 = 2.116

z = 0.51

Prob > |z| = 0.6082

1. Sensitivity analysis for IGRA and TST


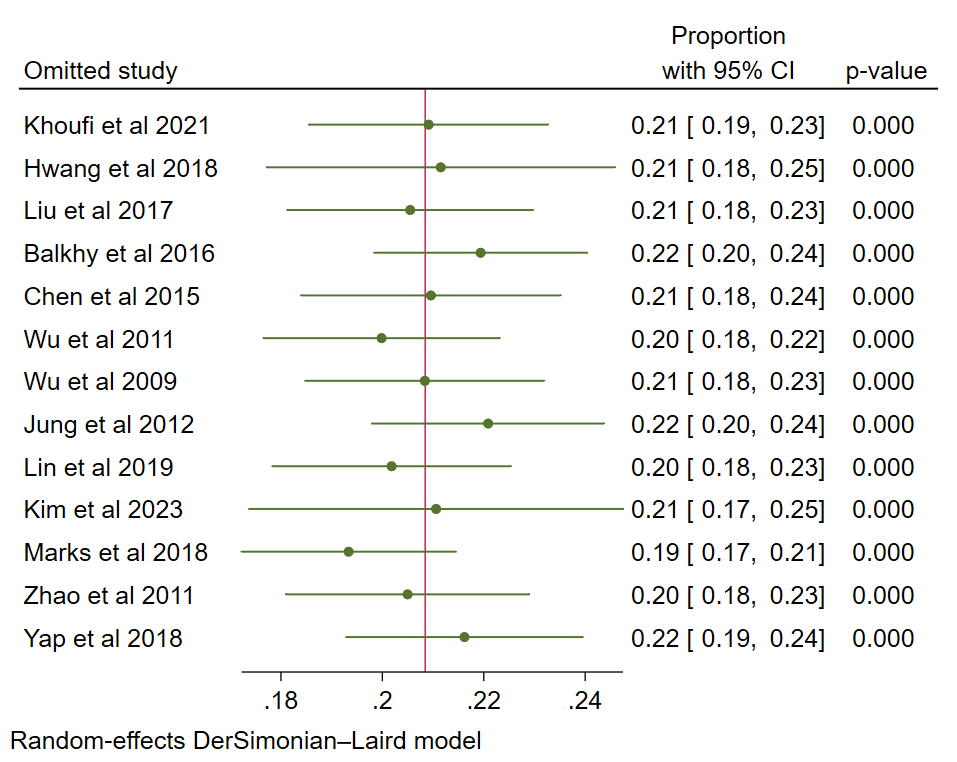

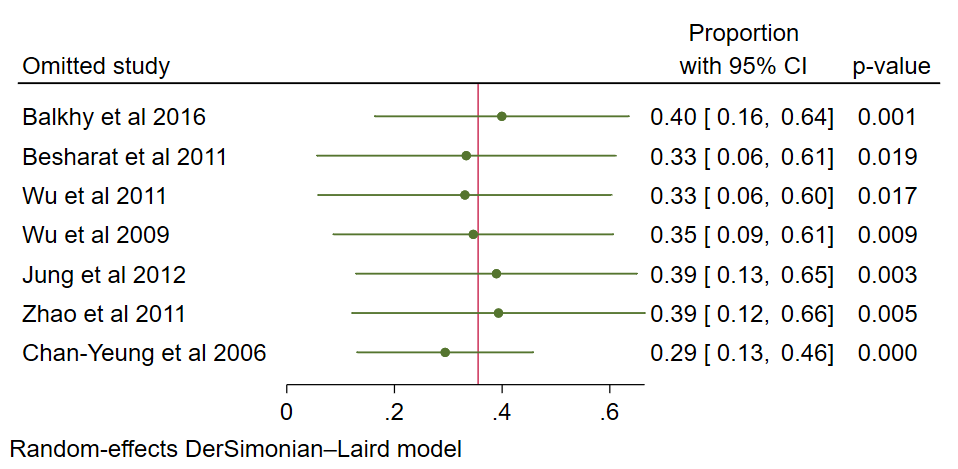

Supplement: Supplementary file 3 — S3: Publication bias, Regression based Eggers’ test and sensitivity analysis for IGRA and TST. [file IID3-12-e1200-s002.docx]
